# Physiotherapy of the Trunk Related to Sitting Function After Stroke: A Delphi Study

**DOI:** 10.1177/02692155251322263

**Published:** 2025-02-28

**Authors:** Elizabeth Bell, Kathy Briffa, James McLoughlin, Robyn Fary

**Affiliations:** 1 Curtin School of Allied Health, 1649 Curtin University, Perth, Australia; 2 College of Nursing and Health Sciences, 1065 Flinders University, Adelaide, Australia; 3 enAble Institute, 1649 Curtin University, Perth, Australia

**Keywords:** Rehabilitation, trunk control, physiotherapy, stroke, sitting balance

## Abstract

**Objective:**

To develop consensus statements from a Delphi panel about physiotherapy of the trunk related to sitting function for people with subacute stroke, with the express aim of facilitating treatment choices by novice physiotherapists.

**Design:**

A four-round e-Delphi study using free-text responses and 5-point Likert scales for agreement.

**Participants:**

Twenty-six panel members with expertise in clinical and/or research in neurological rehabilitation.

**Main measures:**

Round 1 consisted of 5 free-text questions. Subsequent rounds ascertained agreement and consensus on statements formulated from Round 1 responses. Consensus was defined a priori as ≥70% agreement. Round 3 presented an additional two clinical observation queries related to the statements for comment.

**Results:**

Twenty participants completed all four rounds. Nineteen of 26 participants (73%) thought physiotherapy of the trunk was important through all stages of recovery after stroke. Different interpretations about what constitutes physiotherapy of the trunk following stroke were identified. Fourteen statements of agreement regarding physiotherapy of the trunk were formulated. The majority of statements involved different activities in sitting such as control of movement over the base of support and reaching with the unaffected arm. In addition to the statements of agreement clinical observations for implementation of the statements were developed.

**Conclusions:**

In the absence of detail in clinical guidelines and a wide range of interventions in systematic reviews, this study provides clear and specific options for novice physiotherapists of treatment of the trunk related to functional task practice in sitting and as preparation for sit to stand.

## Introduction

Rehabilitation has a fundamental role in facilitating functional recovery and independence after stroke.^1,2^ Trunk performance, has been recognised as an important early predictor of functional outcome after stroke,^3^ and impaired trunk control after stroke is closely associated with poor mobility performance and trunk instability in gait.^4^ Trunk control involves the ability of the muscles across the trunk, pelvis and hip to keep the body upright against gravity, to control weight shifts of the body for various functional movements and to maintain the centre of mass within the base of support during postural adjustments.^5^ As the trunk provides proximal stability for upper and lower limb movement during activities of daily living, impaired trunk control can hinder activity performance and functional independence.^6^ It follows that trunk training after stroke improves sitting and standing balance, dynamic trunk control and mobility,^7–9^ and as such is recognised by clinicians and in the literature as important to the recovery of optimal function in people after stroke.^5,10,11^ The focus of this study was on trunk control in sitting.

Previous systematic reviews and meta-analyses^7,12^ have concluded however, that it is difficult to provide clinical recommendations due to the variety of treatment protocols reported in the different studies. Van Criekinge et al.^7^ recommend future research to examine the type of exercise that is optimal for trunk training in stroke rehabilitation.

Furthermore, different terminology is used, apparently interchangeably, across studies to describe physiotherapy interventions related to the trunk. These include trunk exercises, trunk regimes, trunk treatment, trunk rehabilitation and, most commonly, trunk training.^9,11,13,14^ The term ‘physiotherapy of the trunk’ is therefore used, when appropriate, in this study as an all-inclusive term.

In addition to the lack of clinical recommendations in the literature, national clinical practice guidelines for stroke rehabilitation.^15–18^ are variable and offer limited guidance related to treatment of the trunk. This variability and lack of detail in the guidelines points to a potential inconsistency of practice across settings and makes it particularly challenging for physiotherapists with limited experience, novices, to seek out information on best practice related to physiotherapy of the trunk.

This study examined consensus among a Delphi panel of physiotherapists with expertise in rehabilitation of people with stroke to develop a list of statements about physiotherapy of the trunk for people with subacute stroke. It is proposed that this list of consensus statements could be used by novice physiotherapists to provide more detail to guide treatment of impairments of trunk control in sitting in people with sub-acute stroke (7 days to 6 months post stroke).^19^ Gaining consensus on important elements of physiotherapy for the trunk will facilitate a structured evaluation of practice in clinical and research settings.

## Methods

An e-Delphi study was conducted with the process conforming to the Guidance on Conducting and REporting DElphi Studies.^20^ The study was approved by the Curtin Human Research Ethics Committee (HRE2021-0768).

### Delphi Panel Participants

This study aimed to recruit 20 to 30 physiotherapists with expertise in rehabilitation of people with stroke. This number is within the range considered optimal for these designs.^21,22^ There are no universally agreed selection criteria for ‘experts’ in Delphi studies^21–24^ but recommendations for selection of experts centre around panel members having expertise or experience in a particular field.^22,24^ The inclusion criteria in this study were physiotherapists with at least four years of clinical or research experience in neurological physiotherapy, specifically working with people with stroke. Physiotherapists with the required neurological expertise were identified through professional networks (email distribution lists, closed Facebook groups and snowballing) across Australia and invited to participate via email. If a physiotherapist was unable to accept the invitation, they were asked if they could identify other eligible colleagues in their networks. Twenty-nine physiotherapists from across Australia were invited to participate.

The study invitation contained a link to the participant information statement, consent form and the first round of the Delphi process. Participants were informed of the aim of the project as well as the number of rounds (maximum of 4) and requirements for each round. An estimate of the total time commitment for the process was also provided. Participants gave informed consent at the beginning of the first round survey.

### Data Collection and Analysis

**Figure 1.**
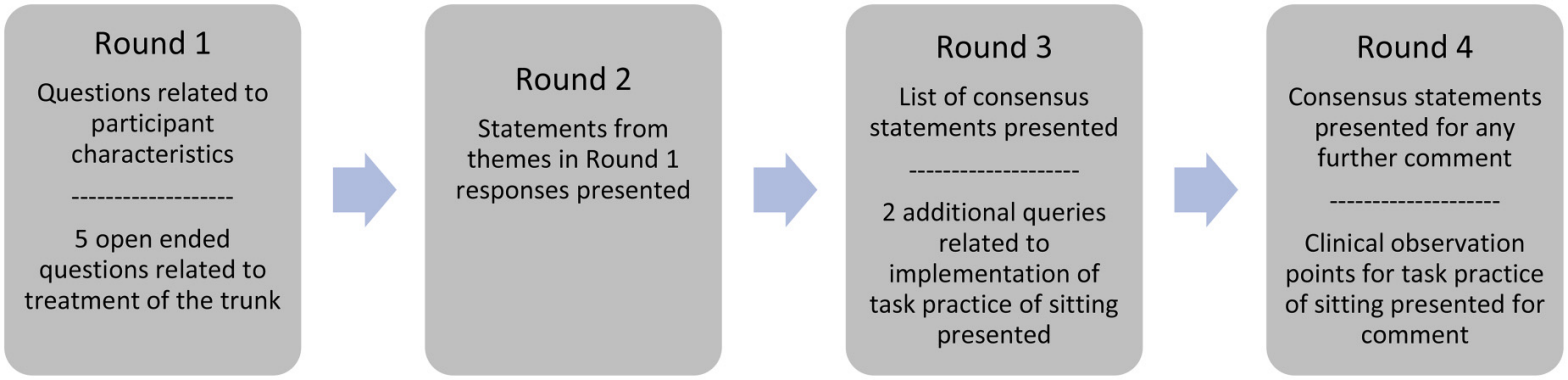
Overview of the four round Delphi process.

Four Delphi rounds were conducted using Qualtrics online surveys (Qualtrics, Provo, UT). Participants were given up to two weeks to respond to each round. One reminder email was sent for each round to participants who had not completed the round allowing a one-week extension to respond Figure 1.

In Round 1, characteristics of the participants (age, clinical experience and academic qualifications) were collected. Participants were also asked to provide answers to five questions related to physiotherapy of the trunk in people in rehabilitation in the subacute phase after stroke. Questions one to three were primary questions and questions four and five were secondary questions for additional information. An essay text box was provided after each question for the response.

The five questions were:

1. When do you think physiotherapy of the trunk is important for people after stroke? Please explain.
2. What activities/exercises/techniques would you use to treat the trunk in people in the subacute phase of recovery after stroke (7 days to 6 months) who are unable to sit without assistance?
3. What activities/exercises/techniques would you use to treat the trunk in people in the subacute phase of recovery after stroke (7 days to 6 months) who are able to sit without assistance?
4. What parameters do you think need to be considered in planning treatment of the trunk for people in rehabilitation in the subacute phase of recovery after stroke?
5. What guidelines would help you decide the parameters for treatment of the trunk for people in rehabilitation in the subacute phase of recovery after stroke?

Responses to each question were analysed using content analysis.^25^ The anonymised raw data was grouped into units of meaning before being condensed into codes, and then grouped into themes by EB. The anonymised raw data and the themes were subsequently reviewed by another member of the research team (JM) to ensure the themes developed were true to the meaning of the raw data.^26^ Lists of statements were developed from these themes and used in Round 2.

In Round 2 participants were asked to rate their agreement with each statement developed in Round 1 using a 5 point Likert scale with the extremes labelled (1 = strongly agree and 5 = strongly disagree) as well as a neutral central point to reduce the risk of respondent uncertainty.^22^ Participants were also able to provide open text comments at the end of each list of statements.

Agreement with each statement was determined by the number of responses of *somewhat agreed and strongly agreed* as a percentage of the total number of responses. Consensus was determined a priori to be reached if ≥ 70% of participants indicated agreement with a statement, this consensus level being supported in the research as adequately rigorous.^22,27,28^ A list of statements of agreement for which consensus was reached was then formulated for use in Round 3.

At this stage it was noted that many of the statements that had reached consensus related to functional task practice of sitting, which is consistent with recommendations in the Australian Stroke Foundation Clinical Guidelines.^17^ However, as the Guidelines do not provide specific advice on implementation, two extra queries seeking opinion about which critical features needed to be observed for implementation of this practice were developed.

Opinion was sought specifically on foot loading and reach distance as these are critical elements to the task practice of sitting as outlined in the seminal article^29^ that underpins the recommendation in the guidelines.^17^

1. Please comment on the clinical observations you consider to indicate loading of the affected foot in sitting practice tasks. This may include some or all of the following: kinematic features at the ankle, knee and hip; kinematic features of movement at the pelvis, spine and thorax; alignment of body segments and foot contact with the floor; plinth height and amount of thigh support; any variations depending on whether or not the person can sit unsupported.
2. For reaching (beyond arm's length) with the unaffected arm in practice of sitting and reaching, what clinical observations do you consider to inform reach distance?

In Round 3, a list of consensus statements was presented, and participants were invited to suggest any changes to these statements using an open text box. In addition, participants were asked to respond to the two extra queries. Comments from these additional queries were collated into categories of observational reference points by EB. The categorised responses were reviewed by another member of the research team (JM) to ensure the categories developed were true to the meaning of the raw data.

In Round 4 the statements of agreement were presented again for any further comments. The categorised responses of observational reference points for functional task practice of sitting were presented to participants for comment.

## Results

Twenty-nine physiotherapists were invited to participate, of which 26 (90%) volunteered for the study.

Participants were from New South Wales (n = 6), Victoria (n = 5), South Australia (n = 3) and Western Australia (n = 12). The median years of clinical experience working with people with stroke was 22 (range 4–45). Almost 70% of respondents had greater than 20 years’ experience working clinically in neurological physiotherapy. Eighty-five per cent of participants were currently working clinically in neurological physiotherapy and 70 per cent had been involved in research in neurological areas.

In response to Question 1, 19 participants (73%) thought physiotherapy of the trunk was important across all stages of recovery after stroke. Five participants (19%) answered that physiotherapy of the trunk was not important/not a priority following stroke. One participant was undecided based on the definition and one participant gave no comment. Example text responses to explain the answers to Question 1 are presented in Table 1.

**Table 1.** Examples of responses to round 1 question 1 “when do you think physiotherapy of the trunk is important for people after stroke? please explain” illustrating the divergent opinion about the importance of trunk treatment during rehabilitation following stroke.

Affirmative example responses
Primarily in the acute and subacute periods after stroke (i.e., day 2–60) Immediately they are medically stable – the trunk provides a framework for the limbs and provides a basis from which perception of verticality and stability can be integrated At all stages after stroke = from acute to rehab and outpatient Immediately after stroke, even if they cannot sit independently. Trunk control and strength is fundamental for stroke recovery. It is required for L/L & U/L control and gait
**Negative example responses**
I don’t think specific treatment of the trunk is important following stroke for a number of reasons. Firstly, the trunk is bilaterally innervated and strength of muscles of the trunk is minimally affected if at all. Secondly, biomechanically the muscles of the trunk can only influence the posture of the trunk but do not control the position of the trunk over the base of support. In other words, muscles of the trunk control the shape/posture of the trunk as they are attached within the trunk. Trunk muscles cannot biomechanically control the trunk relative to the base of support. For example, in sitting, the muscles which are able to control the position of the trunk on the base of support are the muscles of the hip, as well as the knee and ankle (if the foot is on the ground). Specific treatment focused on the trunk is not a priority following stroke. The trunk is largely bilaterally innervated and therefore issues with sitting and standing alignment are rarely related to the ability to recruit muscles of the trunk. More important is the strength of the lower limb. The muscles of the trunk do not cross the hip joint and therefore do not change the angle of the trunk in relation to the femur.

From the themes developed from responses to Question 2, 11 statements were developed. A further nine statements were developed from the themes developed from responses to Question 3. (Table 2) There were no obvious themes in the responses to Question 4 and Question 5. The responses provided suggested that the participants were interpreting the terms “parameters” and “guidelines” used in the questions differently resulting in divergent responses. A definition of “parameter” and examples of expectations were not provided to panellists preventing them from providing more specific and directed responses.

**Table 2.** Levels of agreement with round 2 statements (developed from round 1 questions 2 & 3).

Statement	Percentage agreement	No of responses	Strongly agree (n)	Somewhat Agree (n)	Neither agree nor disagree (n)	Somewhat disagree (n)	Strongly disagree (n)
Question 2 statements: Therapeutic activities used to treat the trunk in people in the sub-acute phase of stroke who are unable to sit without assistance							
*Orientation to midline should be incorporated into supported sitting practice*							
	96	26	21	4	0	1	0
*Targeting loading of the affected foot is desirable in practice of supported sitting*							
	92	26	17	7	1	0	1
*Practicing sitting with environmental support (e.g., next to a wall on the unaffected side) can be useful for improving the function of sitting*							
	88	26	13	10	0	2	1
*Practicing supported sitting and reaching with the unaffected arm beyond arm's length can be useful for improving the function of sitting*							
	84	25	16	5	2	2	0
*Practicing functional tasks such as rolling and supine to sit with recruitment of trunk muscle activity can be useful to train preparation for the function of sitting and transition to/from sitting.*							
	81	26	17	4	0	3	2
*Practicing sitting with hands on support/assistance can be useful to improve the function of sitting*							
	73	26	11	8	1	5	1
*Practicing supported sitting and assisted reaching with the affected arm can be useful for improving the function of sitting*							
	73	26	9	10	2	5	0
*Practicing co-ordinated movement of the upper and lower trunk with assistance in sitting can be useful for improving the function of sitting*							
	73	26	14	5	0	2	5
*Training of trunk muscles in crook lying (e.g., lumbopelvic control; lower trunk rotation) can be useful in preparation for the function of sitting*							
	73	26	13	6	1	1	5
*Restricted passive range of the trunk or neck muscles should be addressed for people in rehabilitation who cannot sit unsupported after stroke*							
	*53*	*26*	*5*	*9*	*5*	*3*	*4*
*Training extensor strength of the affected leg in supine (e.g., practicing pushing on the end of the bed) can be useful for improving the function of sitting*							
	*47*	*26*	*6*	*6*	*9*	*2*	*3*
Question 3 statements: Therapeutic activities used to treat the trunk in people in the sub-acute phase of stroke who are able to sit without assistance							
*Ability to control movement of the trunk over the base of support in sitting is important to the function of sit to stand (Practicing control of movement of the trunk over the base of support is useful in preparation for sit to stand)*							
	100	26	25	1	0	0	0
*Practicing sitting and reaching with the unaffected arm in different directions beyond arm's length can be useful for improving the control of movement of the trunk over the base of support in sitting*							
	92	26	18	6	2	0	0
*Targeting loading of the affected foot should be included in practice of sitting and reaching.*							
	88	26	20	3	1	1	1
*Practicing control of movement of the trunk in different directions over the base of support in sitting can be useful for improving the function of sitting*							
	88	26	19	4	2	1	0
*Practicing coordination of lumbopelvic movements in sitting can be useful for improving the ability to weight transfer to both sides in sitting*							
	73	26	16	3	1	2	4
*Practicing control of movement of the trunk in different directions over the base of support in sitting on an unstable surface can be useful for improving the function of sitting*							
	*69*	*26*	*6*	*12*	*3*	*4*	*1*
*Practicing coordination of upper and lower trunk movements over the base of support in sitting can be useful for improving reaching ability in sitting*							
	*69*	*26*	*12*	*6*	*2*	*3*	*3*
*Restricted passive range of the trunk or neck muscles should be addressed for people in rehabilitation who are able to sit unsupported after stroke*							
	*57*	*26*	*6*	*9*	*6*	*2*	*3*
*Practicing coordination of lumbopelvic movement in 4-point kneeling can be useful for improving the control of movement of the trunk over the base of support in sitting*							
	*46*	*26*	*2*	*10*	*7*	*2*	*5*

All 26 participants completed the Round 2 survey.

Over 70% of respondents agreed or strongly agreed with nine of the 11 statements developed from Round 1, Question 2. Over 70% of respondents agreed or strongly agreed with five of the nine statements developed from Round 1, Question 3. (Table 2) Most of the statements that reached consensus involved activities in sitting (e.g., with control of movement of the trunk over the base of support, with reaching of the arm, and with practice of coordination of lumbopelvic movements). Factors such as orientation to midline and loading of the foot were considered important for activities in sitting. Practicing control of movement of the trunk over the base of support in sitting was considered important not only to improve the function of sitting but also as preparation for sit to stand (100% agreement). Only two consensus statements were not activities in sitting: practice of rolling and supine-to-sit with recruitment of trunk muscle activity, and training of trunk muscles in crook lying (e.g., lumbopelvic control, lower trunk rotation). Both were for people who were unable to sit without assistance. A list of consensus statements is presented in Table 3.

**Table 3.** Consensus statements of therapeutic activities for treatment of the trunk.

For people in the subacute phase of stroke who are unable to sit unsupported	% agreement
Orientation to midline should be incorporated into supported sitting practice	96
Targeting loading of the foot is desirable in practice of supported sitting	92
Practicing sitting with environmental support (e.g., next to a wall on the unaffected side) can be useful for improving the function of sitting	88
Practicing supported sitting and reaching with the unaffected arm beyond arm's length can be useful for improving the function of sitting.	84
Practicing functional tasks such as rolling and supine to sit with recruitment of trunk muscles (can be useful to train preparation for the function of sitting and transition to/from sitting)	81
Practicing sitting with hands on support/assistance can be useful for improving the function of sitting	73
Practicing supported sitting and assisted reaching with the affected arm can be useful for improving the function of sitting	73
Practicing co-ordinated movement of the upper and lower trunk with assistance in sitting can be useful for improving the function of sitting	73
Training of muscles in crook lying (e.g., lumbopelvic control; lower trunk rotation) can be useful in preparation for the function of sitting	73
**For people in the subacute phase of stroke who are able to sit unsupported**	% agreement
Practicing control of movement of the trunk over the base of support in sitting is useful in preparation for sit to stand	100
Practicing sitting and reaching with the unaffected arm in different directions beyond arms’ length can be useful for improving control of movement of the trunk over the base of support in sitting	92
Targeting loading of the affected foot should be included in the practice of sitting and reaching	88
Practicing control of movement of the trunk in different directions over the base of support in sitting can be useful for improving the function of sitting	88
Practicing co-ordination of lumbopelvic movements in sitting can be useful for improving the ability to weight transfer to both sides in sitting	73

In Round 3, 22 participants (85%) completed the survey. No changes regarding the statements of agreement were suggested.

There were 21 responses to each of the extra queries related to clinical observations for functional sitting tasks. All responses were represented within categories of critical observation points for each query (Table 4).

**Table 4.** Clinical observations for implementation of sitting practice.

Please comment on the clinical observations you consider to indicate loading of the affected foot in sitting practice tasks
**Categorisation of response comments:**
**Kinematics of hip, knee and ankle**
**Ankle**
Feet in contact with the floor (step if feet don’t reach floor) Flat foot contact; ankle at least 90 degrees/slight dorsiflexion; feet positioned underneath knee; all toes in contact with the ground; observation of proprioceptive contact; evidence of foot activity. Feet roughly hip width apart **Hip and knee** Hip and knee at least 90 degrees; approx. 105 degrees knee flexion. Feet positioned underneath knees; hips higher than knees; hip in neutral abduction/adduction/rotation. Hips higher than knees and tending towards ant tilt. Height of the seat determines angle of ankle, hip and knee, hip and knee angle will be determined by the height of the surface the person is sitting on, as well as their leg length. **Kinematic features of movement at the pelvis, spine, and thorax** Trunk upright extension through lumbar spine and thorax; degree of extension at the pelvis and hemi-trunk Movement of the pelvis, head in neutral alignment, spine and thorax should be achieved with the foot loaded; Continued foot support with surface during movement at pelvis e.g., pelvic tilt ant/posterior, lateral movement of pelvis/trunk. Activity observed around hip/pelvis. **Alignment of body segments** Optimising symmetry between left and right where able, oriented to midline; alignment and direction of the trunk in midline. Alignment of the hip, knee and ankle; sitting in alignment with spine over pelvis; whether there is a relationship between the alignment and the task being executed. Position of the head in relationship to the trunk and pelvis **Plinth height and amount of thigh support** According to ability – higher plinth, less thigh support for more challenge Amount of thigh support should be adjusted according to ability i.e., more thigh support to make it easier to sit and decrease thigh support to increase the difficulty/increase the loading on affected foot. Plinth height is important as it can make loading during an activity easier or harder. Set up variables for difficulty, progression of task, plinth height, thigh support BOS. Height of the plinth has to allow foot to be placed on floor. Is the amount of thigh support optimal to promote weight bearing?
**For reaching (beyond arm's length) with the unaffected arm in practice of sitting and reaching, what clinical observations do you consider to inform reach distance?**
Categorisation of response comments **Trunk movement observations** Lateral tilt and extension of the trunk/lengthening of the trunk through the unaffected side Control of movement of the trunk in direction of reach **Loading of the leg/foot movement observations** Loading of the leg/foot in the direction of the reach The ability to tilt the pelvis and maintain foot contact with the floor whilst reaching with the unaffected arm. Are they loading the leg in the direction of the reach and further is there palpable mm activity in their leg extensors Is reach through stabilisation activity of the foot, leg and pelvis supporting thorax and shoulder towards antigravity direction or is it through lateral flexion of the trunk. **Reach distance observations** Distance reached whilst maintaining trunk extension. How far can the person reach beyond arm's length without compensations? Height more than lateral displacement Usually expressed as rough % arm's length Consider the features that modulate difficulty - these include distance but also direction, height, timing/task used.

In Round 4, 20 participants (77%) completed the survey. Eight participants (40%) made further comment on the statements of agreement; four participants (20%) made a comment related to the clinical observational points for sitting functional tasks (loading of the foot); and five participants (25%) made a comment related to the clinical observational points for sitting functional tasks (reaching distance). Nine participants (45%) made no further comment for the statements of agreement or the clinical observation points.

Most comments (75%) that related to the statements of agreement concurred with the statements, with two participants (25%) expressing concerns about the list not being evidenced based.

For comments related to the clinical observational points one participant disagreed with the terminology used relating to trunk and pelvis, with all other comments (eight participants) being in agreement.

## Discussion

This Delphi study gathered consensus opinion on physiotherapy of the trunk related to sitting function for people with subacute stroke.

When asked when physiotherapy of the trunk is important after stroke some participants in this study thought it was either not important or not a priority after stroke. Not having a universally accepted understanding of the phrase ‘physiotherapy of the trunk’ could account for this result. Some participants interpreted the term as training/treatment that only applied to those muscles within the trunk region that influence posture of the trunk whereas other participants appeared to interpret trunk training as incorporating muscles of the pelvis and hip as well as the trunk. Interestingly, opinion about when physiotherapy is important after stroke did not differ by length of time as a physiotherapist working with people with stroke. Nevertheless, despite differences in interpretation, the majority of participants in this study thought physiotherapy of the trunk was important at all stages of recovery after stroke and 14 statements of agreement were formulated. Twelve of the 14 statements involved some form of functional task practice both for people who were able and unable to sit unsupported.

This outcome is consistent with the reported effectiveness of task practice in rehabilitation after stroke.^30–31^ Describing activities as functional task practice in sitting presents an alternative to use of the general term ‘trunk training ‘and a way forward for consistency in clinical practice. Further research to describe the selective movement of the trunk and pelvis involved in moving over the base of support would add further detail to guide implementation of task practice. For example, in moving the trunk forward over the base of support in sitting describing whether the movement is predominately achieved with head and trunk flexion or by hip flexion with lumbar extension is relevant as it creates a different relationship between movement of the centre of mass and the base of support.

Clinical guidelines for stroke rehabilitation^15–18^ currently provide limited guidance for physiotherapy of the trunk after stroke. A recommendation for “trunk training exercises” is included in the UK and Canadian guidelines but these are non-specific.^15,16^ The practice of sitting and reaching with the unaffected arm is the only recommendation in relation to rehabilitation for people who have difficulty sitting in the Australian^17^ and Dutch^18^ guidelines. The seminal study^29^ upon which this recommendation is based excluded patients with a visual problem or any major cognitive or perceptual problems. Patients also needed to be able to sit unsupported (a score of at least 3 on the Motor Assessment Scale for Stroke), be able to reach a distance equivalent to 140% of arm's length with the intact arm and understand instructions.^29^ For a novice physiotherapist, application of this guideline with a patient who is unable to sit unsupported and perhaps has neglect or cognitive impairment maybe challenging. Consequently, caution is required when generalising these findings to patients who do not meet these selection criteria.

This Delphi study also gathered opinion on clinical observation points for the task practice of sitting. These points may assist in standardising the execution of activities in sitting. For example, what a clinician should observe to check for optimal loading of the affected foot in the task practice of sitting and reaching with the unaffected arm, as recommended in the Stroke Foundation Clinical Guidelines.^17^

As reported by the Inaugural Stroke Rehabilitation and Recovery Roundtable, incomplete descriptions of interventions reported in research hinder the usability and development of standardised rehabilitation interventions.^32^ Therefore, the consensus statements from this study provide a detailed list of activities deemed relevant for current practice in physiotherapy of the trunk in people with sub-acute stroke. This information can also potentially be used to report on trunk control interventions in observational and comparative studies of physiotherapy after stroke.

A major strength of this study was the high response rates across Delphi rounds (100% to 77%), indicating a high level of engagement with this topic. Also 18 of the 26 participants (69%) had over 20 years of experience working in neurological physiotherapy highlighting the depth of expertise within the panel. Further, the number of participants recruited was acceptable according to the Delphi literature and 85% of respondents were currently working clinically and were representative of contemporary and experienced clinical practice.

It is acknowledged that not all Australian states were represented in this study. As this study only sought input from physiotherapists within Australia, the findings may not translate to other countries and other health systems.

In conclusion, in the absence of comprehensive guidelines for current practice of physiotherapy of the trunk related to sitting function the list of consensus statements produced in this Delphi study can guide novice physiotherapists in treatment choices when addressing impairments in trunk control and sitting function in people with subacute stroke.

We know there is evidence that supports the predictive value of early sitting balance in improving functional outcome after stroke,^3^ but the optimal treatment to achieve improved sitting balance is unclear. Highlighting the commonality in opinion on the importance of task practice to the function of sitting in this study may serve as a starting point to increase clarity around physiotherapy of the trunk after stroke. Perhaps a reframing of language to ‘treatments to improve sitting function’ and future research that attempts to clarify treatments that improve movement control of the trunk over the base of support in sitting is a worthwhile direction.

**Clinical Message**

- Physiotherapists with expertise in stroke rehabilitation agree that a range of activities in sitting can be used to improve sitting function of people in the subacute phase of stroke and that orientation to the midline and loading of the foot are considered important in the practice of activities in sitting.
- In addition, control of movement of the trunk over the base of support in sitting is considered important to the function of sit to stand.
- The statements created provide detail to guide treatment choices by novice physiotherapists.

## References

[1] Winstein CJ Stein J Arena R , et al. Guidelines for adult stroke rehabilitation and recovery: a guideline for healthcare professionals from the American Heart Association/American stroke association. Stroke 2016; 47: e98–e169. 10.1161/STR.0000000000000098 27145936

[2] Enderby P Pandyan A Bowen A , et al. Accessing rehabilitation after stroke - a guessing game? Disabil Rehabil 2017; 39: 709–713. 27133783 10.3109/09638288.2016.1160448

[3] Verheyden G Nieuwboer A De Wit L , et al. Trunk performance after stroke: an eye catching predictor of functional outcome. J Neurol Neurosurg Psychiatry 2007; 78: 694–698. 17178824 10.1136/jnnp.2006.101642 PMC2117706

[4] Isho T Usuda S . Association of trunk control with mobility performance and accelerometry-based gait characteristics in hemiparetic patients with subacute stroke. Gait Posture 2016; 44: 89–93. 27004638 10.1016/j.gaitpost.2015.11.011

[5] Gjelsvik BEB . The Bobath Concept in Adult Neurology. 2nd edition. Stuttgart, Germany: Thieme, 2016.

[6] Pompeu SMAA Pompeu JE Rosa M , et al. Correlation between motor function, balance and respiratory muscular strength after stroke. Revista Neurociências 2011; 19: 614–620.

[7] Van Criekinge T Truijen S Schroder J , et al. The effectiveness of trunk training on trunk control, sitting and standing balance and mobility post-stroke: a systematic review and meta-analysis. Clin Rehabil 2019; 269215519830159: 1–11. 10.1177/0269215519830159 30791703

[8] Cabanas-Valdes R Cuchi GU Bagur-Calafat C . Trunk training exercises approaches for improving trunk performance and functional sitting balance in patients with stroke: a systematic review. NeuroRehabilitation 2013; 33: 575–592. 24018373 10.3233/NRE-130996

[9] Alhwoaimel N Turk R Warner M , et al. Do trunk exercises improve trunk and upper extremity performance, post stroke? A systematic review and meta-analysis. NeuroRehabilitation 2018; 43: 395–412. 30400112 10.3233/NRE-182446

[10] Kilinc M Avcu F Onursal O , et al. The effects of bobath-based trunk exercises on trunk control, functional capacity, balance, and gait: a pilot randomized controlled trial. Top Stroke Rehabil 2016; 23: 50–58. 26260878 10.1179/1945511915Y.0000000011

[11] Karthikbabu S Chakrapani M Ganesan S , et al. Efficacy of trunk regimes on balance, mobility, physical function, and community reintegration in chronic stroke: a parallel-group randomized trial. J Stroke Cerebrovasc Dis 2018; 27: 1003–1011. 29361348 10.1016/j.jstrokecerebrovasdis.2017.11.003

[12] Moreno-Segura N Martin-San Agustin R Garcia-Bafalluy S , et al. Effects of core training on trunk function, balance, and gait in stroke patients: A systematic review and meta-analysis of randomised controlled trials. Clin Rehabil 2022; 2692155221117220: 1–20. 10.1177/02692155221117220 35892183

[13] Van Criekinge T Saeys W Vereeck L , et al. Are unstable support surfaces superior to stable support surfaces during trunk rehabilitation after stroke? A systematic review. Disabil Rehabil 2018; 40: 1981–1988. 28482696 10.1080/09638288.2017.1323030

[14] Souza DCB de Sales Santos M da Silva Ribeiro NM , et al. . Inpatient trunk exercises after recent stroke: an update meta-analysis of randomized controlled trials. NeuroRehabilitation 2019; 44: 369–377. 31177237 10.3233/NRE-182585

[15] Teasell R Salbach NM Foley N , et al. Canadian Stroke best practice recommendations: rehabilitation, recovery, and community participation following stroke. Part one: rehabilitation and recovery following stroke; 6th edition update 2019. Int J Stroke 2020; 15: 763–788. 31983296 10.1177/1747493019897843

[16] National Clinical Guideline for Stroke. 5th edition. London: NaNRoyal College of Physicians, 2016.

[17] Stroke Foundation. Clinical guidelines for stroke management (2023).

[18] KNGF Clinical Practice Guideline for Physical Therapy in patients with stroke (2014)

[19] Bernhardt J Hayward KS Kwakkel G , et al. Agreed definitions and a shared vision for new standards in stroke recovery research: the stroke recovery and rehabilitation roundtable taskforce. Int J Stroke 2017; 12: 444–450. 28697708 10.1177/1747493017711816

[20] Junger S Payne SA Brine J , et al. Guidance on conducting and REporting DElphi studies (CREDES) in palliative care: recommendations based on a methodological systematic review. Palliat Med 2017; 31: 684–706. 28190381 10.1177/0269216317690685

[21] Nasa P Jain R Juneja D . Delphi methodology in healthcare research: how to decide its appropriateness. World J Methodol 2021; 11: 116–129. 34322364 10.5662/wjm.v11.i4.116 PMC8299905

[22] Drumm S Bradley C Moriarty F . ‘More of an art than a science'? The development, design and mechanics of the delphi technique. Res Social Adm Pharm 2022; 18: 2230–2236. 34244078 10.1016/j.sapharm.2021.06.027

[23] Keeney. Consulting the oracle ten lessons from using the Delphi technique in nursing. J Adv Nurs 2006; 53: 205–212. 16422719 10.1111/j.1365-2648.2006.03716.x

[24] Sablatzky T . Methods moment: The delphi method. Hypothesis (Macon) 2022; 34: 1–6.

[25] Kleinheksel AJ Rockich-Winston N Tawfik H , et al. Demystifying content analysis. Am J Pharm Educ 2020; 84: 7113. 32292185 10.5688/ajpe7113 PMC7055418

[26] Keeney S McKenna H Hasson F . The Delphi Technique in Nursing and Health Research. Chicester, UNITED KINGDOM: John Wiley & Sons, Incorporated, 2011.

[27] Metzelthin SF Rostgaard T Parsons M , et al. Development of an internationally accepted definition of reablement: a delphi study. Ageing Soc 2020; 42: 703–718.

[28] Shang Z . Use of delphi in health sciences research: a narrative review. Medicine (Baltimore) 2023; 102: e32829. 10.1097/MD.0000000000032829 PMC9936053 36800594

[29] Dean CM Channon EF Hall JM . Sitting training early after stroke improves sitting ability and quality and carries over to standing up but not to walking: a randomised controlled trial. Australian Journal of Physiotherapy 2007; 53: 97–102. 17535145 10.1016/s0004-9514(07)70042-9

[30] Maier M Ballester BR Verschure P . Principles of neurorehabilitation after stroke based on motor learning and brain plasticity mechanisms. Front Syst Neurosci 2019; 13: 74. 31920570 10.3389/fnsys.2019.00074 PMC6928101

[31] Chan BK Ng SS Ng GY . A home-based program of transcutaneous electrical nerve stimulation and task-related trunk training improves trunk control in patients with stroke: a randomized controlled clinical trial. Neurorehabil Neural Repair 2015; 29: 70–79. 24795163 10.1177/1545968314533612

[32] Walker MF Hoffmann TC Brady MC , et al. Improving the development, monitoring and reporting of stroke rehabilitation research: consensus-based core recommendations from the stroke recovery and rehabilitation roundtable. Int J Stroke 2017; 12: 472–479. 28697706 10.1177/1747493017711815

